# Zertifiziertes Weiterbildungscurriculum für die Facharztweiterbildung in Urologie der Deutschen Gesellschaft für Urologie gemäß der Musterweiterbildungsordnung 2018 (Fassung vom 20.09.2019)

**DOI:** 10.1007/s00120-020-01367-7

**Published:** 2020-11-03

**Authors:** M. S. Michel, M. Himmler, U. Necknig, M. Kriegmair, T. Speck, J. Fichtner, J. Steffens, H. Borgmann, C. Bolenz, M. Tuellmann, S. Ruppin, F. Petersilie, U. Rebmann, J. König, J. Westphal, P. Goebell, H. Leyh, H. Borchers

**Affiliations:** 1 Klinik für Urologie und Urochirurgie, Universitätsmedizin Mannheim, Universität Heidelberg, Theodor-Kutzer-Ufer 1–3, 68167 Mannheim, Deutschland; 2 Abteilung für Urologie und Kinderurologie, Klinikum Garmisch-Partenkirchen, Auenstraße 6, 82467 Garmisch-Partenkirchen, Deutschland; 3 Urologische Klinik München Planegg, Germeringer Str. 32, 82152 Planegg, Deutschland; 4 Treskow Allee 103, 10318 Berlin, Deutschland; 5 Klinik für Urologie, Johanniter-Krankenhaus Oberhausen, Steinbrinkstraße 96a, 46245 Oberhausen, Deutschland; 6 Klinik für Urologie und Kinderurologie, St.-Antonius Hospital, Akademisches Lehrkrankenhaus der RWTH Aachen, Dechant-Deckers-Straße 8, 52249 Eschweiler, Deutschland; 7 Klinik und Poliklinik für Urologie und Kinderurologie, Universitätsmedizin Mainz, Langenbeckstr. 1, 55131 Mainz, Deutschland; 8 Klinik für Urologie und Kinderurologie, Universitätsklinikum Ulm, Albert-Einstein-Allee 23, 89081 Ulm, Deutschland; 9 Dr.-Henkel-Str. 2, 85435 Erding, Deutschland; 10 Friedrichstr. 94, 10117 Berlin, Deutschland; 11 Deutsche Gesellschaft für Urologie, Martin-Buber-Str. 10, 14163 Berlin, Deutschland; 12 Große Nikolaistr. 1, 06108 Halle, Deutschland; 13 Abteilung für Urologie, Asklepios Stadtklinik Bad Tölz, Schützenstraße 15, 83646 Bad Tölz, Deutschland; 14 Klinik für Urologie und Kinderurologie, Krankenhaus Maria Hilf der Alexianer Krefeld GmbH, Dießemer Bruch 81, 47805 Krefeld, Deutschland; 15 Urologische und Kinderurologische Universitätsklinik, Malteser Waldkrankenhaus St. Marien, Rathsberger Straße 57, 91054 Erlangen, Deutschland

## Hintergrund und Einleitung

Im Jahr 2012 beauftragte die Bundesärztekammer alle medizinischen Fachgesellschaften in Deutschland mit dem Entwurf einer neuen Musterweiterbildungsordnung (MWBO). Die vorhergehende Version der MWBO stammte aus dem Jahr 2003 und enthielt einige Anforderungen, die einer zeitgemäßen Anpassung bedurften. Die Deutsche Gesellschaft für Urologie (DGU) nutzte ihre einmalige Chance, die Weiterbildung aktiv mitzugestalten und neue Konzepte einzuarbeiten, um so die Aus- und Weiterbildung für Urologie in Deutschland an die veränderten Strukturen und Anforderungen anzupassen und sie dadurch homogener, besser und zukunftsorientierter gestalten. Nach Erarbeitung der neuen MWBO in Zusammenarbeit mit dem Berufsverband (BvDU), sowie der German Society of Residents in Urology (GeSRU) und der Vorstellung der neuen MWBO auf Kongressen und dem Deutschen Ärztetag, wurde diese harmonisiert und schließlich im November 2018 verabschiedet [1]. Bei Betrachtung der Inhalte der neuen Weiterbildungsordnung wird deutlich, dass zum einen – wie auch in anderen Fachgebieten – eine klare Verlagerung der geforderten Kenntnisse und Fähigkeiten in Richtung der ambulanten Medizin stattgefunden hat, zum anderen die tatsächliche Breite des Fachgebiets nun besser abgebildet wird. Die empfohlenen Mindestzahlen wurden reduziert und unterstreichen den Schwerpunkt des breiteren Erwerbs von Fähigkeiten und Kenntnissen weg von einem konstruierten Operationskatalog – Kompetenz statt Quantität ist eine propagierte Errungenschaft der neuen MWBO [2].

Die MWBO stellt lediglich eine Art übergeordnete Empfehlung dar, die mit konkreten Weiterbildungsinhalten entsprechend einem fachlich empfohlenen Weiterbildungsplan (FEWP) gefüllt werden muss. In Deutschland obliegt den Landesärztekammern die Hoheit über die Weiterbildung, weshalb es bundesweit eine gewisse Heterogenität in Bezug auf Weiterbildungsinhalte und -möglichkeiten gibt. Um die Weiterbildungsinhalte homogener gestalten zu können, rief die DGU eine Weiterbildungskonferenz ins Leben, die sich mit der Entwicklung des FEWP im Sinne einer Empfehlung für die Landesärztekammern befasste. Hierfür galt es, für alle vorgegebenen Weiterbildungsaspekte in der MWBO (im Fachgebiet Urologie) die zugehörige „Kognitive- und Methodenkompetenz“, sowie die „Handlungskompetenz“ mit konkreten Inhalten zu füllen.

Im Rahmen der Konkretisierung des fachlich empfohlenen Weiterbildungsplans nach der MWBO von 2018 (Fassung vom 20.09.2019) nahm die DGU die Gelegenheit wahr und entwickelte in einer Weiterbildungskonferenz in Kooperationspartnerschaft mit dem BvDU und der GeSRU ein DGU-zertifiziertes Weiterbildungscurriculum, das im Folgenden vorgestellt wird.

## Das DGU-zertifizierte Weiterbildungs-Curriculum nach der neuen MWBO von 2018 (Fassung vom 20.09.2019)

**Figure 1:**
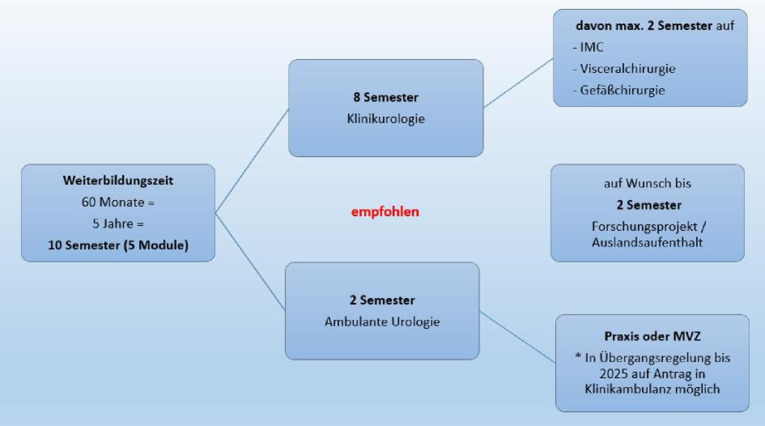

Die Mindestweiterbildungszeit für den Facharzt für Urologie beträgt auch nach der neuen Weiterbildungsordnung weiterhin 60 Monate (5 Jahre). Es bietet sich eine Unterteilung in Halbjahre an, so dass dann insgesamt 10 Semester zur Vermittlung der Weiterbildungsinhalte zur Verfügung stehen. Eine der größten Veränderungen in der MWBO stellt die klare Verlagerung der geforderten Kompetenzen in den ambulanten Bereich der Urologie dar. Dieser Veränderung sollte auch in der Umsetzung der MWBO Rechnung getragen werden. Deshalb empfiehlt das Curriculum, 2 Semester im ambulanten Sektor zu absolvieren, z. B. durch eine Rotation in eine Praxis oder ein medizinisches Versorgungszentrum (MVZ). Dafür ist es notwendig, Kooperationen und Allianzen zwischen den Akteuren zu bilden, im Rahmen derer den Weiterbildungsassistenten entsprechende intersektorale Rotationen ermöglicht werden können. Da solche Veränderungen bis zur finalen Umsetzung zumeist einiger Zeit bedürfen, soll eine Praxisrotation durch eine Übergangsregelung bis 2025 auch in Klinikambulanzen möglich sein. Die verbleibenden 8 Semester sollten im Bereich der stationären Urologie abgeleistet werden. Dabei können fakultative Rotationen von maximal 2 Semestern auf eine Wachstation (IMC), die Viszeral- oder die Gefäßchirurgie angeboten werden (Abb. 1). Hier könnte ein Austausch von Weiterbildungsassistenten zwischen Universitätskliniken/Kliniken mit voller Weiterbildungsermächtigung und Schwerpunkt- und Regelversorgern erfolgen, wenn die entsprechenden Semester beispielsweise im eigenen Haus nicht angeboten werden können. Auf Wunsch des Assistenzarztes können 2 Semester zur Bearbeitung eines speziellen Forschungsprojekts genutzt werden, wodurch sich jedoch die Weiterbildungszeit, je nach Anerkennungsmöglichkeiten an der entsprechenden Klinik, gegebenenfalls verlängern kann. Auf diese Weise kann eine volle und individuell gestaltete Weiterbildung, evtl. wie beschrieben auch im Rotationsaustauschprogramm mit anderen urologischen Kliniken und Praxen, angeboten werden.

**Figure 2:**
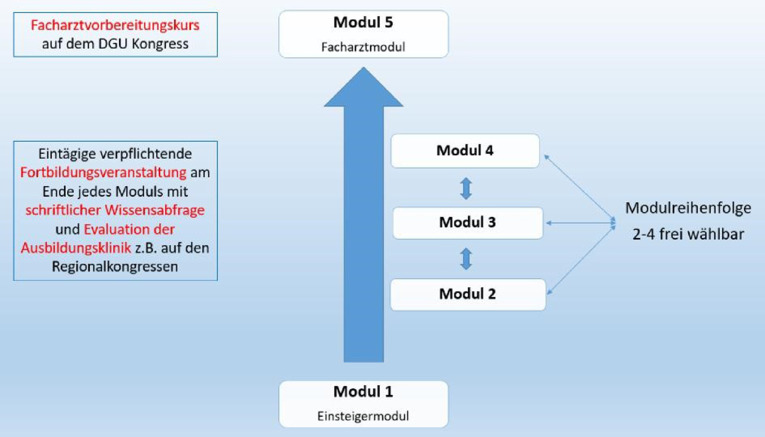

Während der gesamten Weiterbildungszeit werden insgesamt 10 Semester durchlaufen. Fasst man hiervon jeweils 2 Semester (also 1 Weiterbildungsjahr) zusammen, so ergeben sich daraus 5 Module, in denen die Weiterbildungsinhalte vermittelt werden können. Die einzelnen Module sollten individuell so konzipiert sein, dass sie in Summe alle durch die MWBO vorgegebenen Schwerpunkte der Weiterbildung abbilden. Modul 1 stellt dabei das sog. Einsteigermodul dar, das immer als erstes durchlaufen werden sollte und in dem – neben einigen speziellen Inhalten – v. a. grundlegende Kenntnisse und Fähigkeiten der Urologie vermittelt werden sollen. Die Module 2–4 können im Anschluss in ihrer Reihenfolge vom Weiterbildungsassistenten frei gewählt und in ihren Inhalten den Schwerpunkten des Arbeitsplatzes, beispielsweise auch im Rahmen einer Rotation, angepasst werden. Modul 5 sollte von allen Weiterbildungsassistenten als letztes Modul durchlaufen werden und eine optimale Vorbereitung auf die Facharztprüfung ermöglichen (Abb. 2). Um die regelmäßige fachliche Fortbildung der Weiterbildungsassistenten zu unterstützen und zu fördern, ist am Ende jedes Moduls eine eintägige Fortbildung vorgesehen, an deren Ende eine schriftliche (Module 1–4) oder mündliche (Modul 5, Facharztvorbereitungskurs) Wissensabfrage erfolgt. Diese modulspezifischen Fortbildungen könnten vorzugsweise im Rahmen der großen deutschen urologischen Kongresse stattfinden (UroAktuell, DGU-Kongress, evtl. Regionalkongresse). Unabhängig davon wird selbstverständlich der Besuch zusätzlicher Fortbildungsveranstaltungen begrüßt. Im Rahmen der modulspezifischen Fortbildungen können zudem die Ausbildungskliniken von den Weiterbildungsassistenten evaluiert werden.

**Figure 3:**
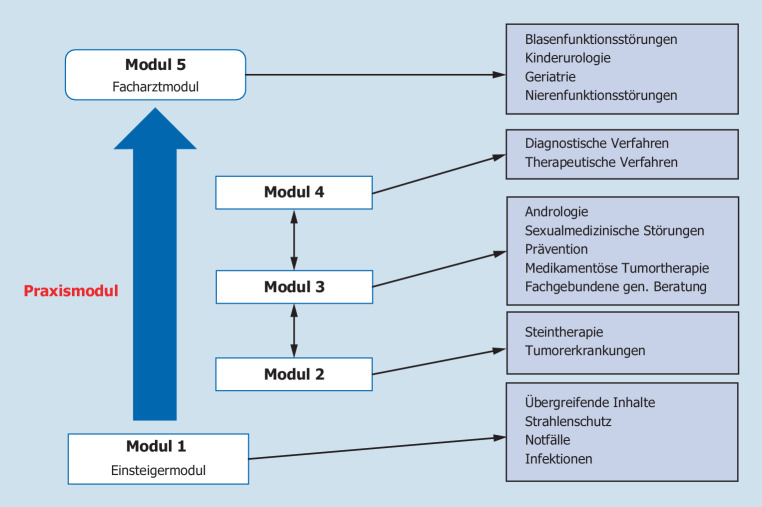

**Figure 4:**
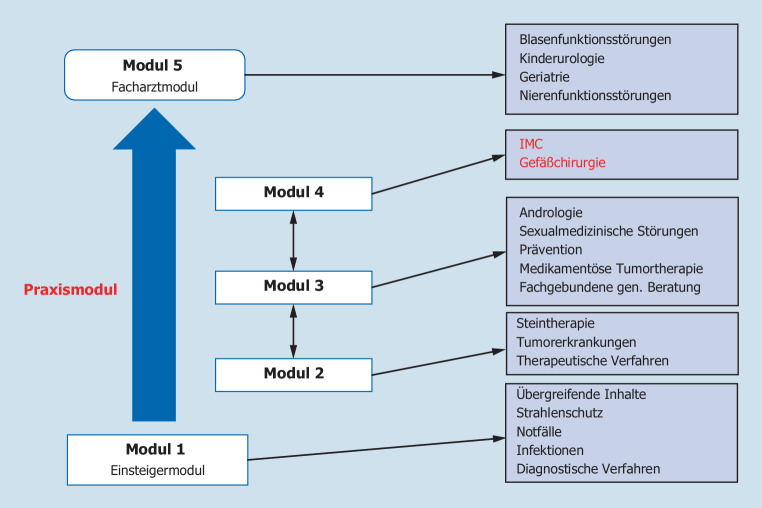

In Abb. 3 und 4 sind mögliche Modelle dargestellt, wie sie im Rahmen eines individuellen Konzepts für einen Weiterbildungsassistenten erarbeitet werden könnten. Hierbei wird deutlich, dass je nach der individuellen Planung des Assistenten und dem Angebot der Weiterbilder sehr unterschiedliche Konzepte möglich sind. Wenn wie in Abb. 4 zwei Semester mit IMC und gefäßchirurgischer Rotation geplant sind, dann müssen die urologischen Weiterbildungsinhalte entsprechend auf die anderen Module verteilt werden, damit zum Facharzt alle erforderlichen Kompetenzen vermittelt und alle Fähigkeiten erlernt werden können. Die einzelnen geforderten Weiterbildungsinhalte sind dabei als übergreifende Schwerpunktthemen zu begreifen, deren Vermittlung sicherlich in vielen Teilen parallel oder überlappend erfolgen kann. Es sollte jedoch ein Augenmerk insbesondere auf speziellere Inhalte gelegt werden, deren Kompetenz nicht unbedingt im üblichen Setting einer Klinik erworben werden kann (beispielsweise Kinderurologie, Urogynäkologie oder Andrologie).

Den am zertifizierten DGU-Curriculum teilnehmenden Kliniken und Praxen wird für die Zertifizierung der Ausbildungsstätten der Weiterbildungsbeauftragte der DGU als Begleiter zur Seite gestellt. Zu Beginn der Teilnahme am DGU-zertifizierten Curriculum wird er in den Kliniken und Praxen das Initiierungsaudit durchführen. Alle 5 Jahre findet dann ein Re-Audit mit Besichtigung und Begehung der Weiterbildungsstätten statt, um sicherzustellen, dass die Kriterien, die für eine hochqualitative und strukturierte Ausbildung erforderlich sind, auch weiterhin erfüllt werden. Auf diese Weise soll die Eignung zur Weiterbildung nicht mehr wie bisher nur von vorgegebenen, zu erfüllenden Mindestzahlen an Behandlungen und Eingriffen abhängen, sondern transparent und objektivierbar anhand nachvollziehbarer Kriterien bescheinigt werden. Im Falle von zwischenzeitlich auftretenden Fragen oder Problemen kann jederzeit ein zusätzliches Interventionsaudit erfolgen. Im elektronischen Logbuch (E-Logbuch), das bundesweit im Rahmen der MWBO vorgesehen ist, werden die Erfüllung der Richtwerte und der Erwerb von Kompetenzen und Fähigkeiten der einzelnen Weiterbildungsinhalte entsprechend den Blöcken der Module sowohl von den Weiterbildungsassistenten, als auch von den Weiterbildern dokumentiert.

Auf einem Portal der DGU wird zur besseren Transparenz eine Auflistung der Kriterien zu finden sein, die für eine Teilnahme am Curriculum vorausgesetzt werden. Dort werden auch die am Programm partizipierenden Kliniken aufgeführt werden. Dies erleichtert es den teilnehmenden Kliniken und Praxen, passende Kooperationspartner zu finden. Die Weiterbildungsassistenten können gemäß ihren Interessen oder dem als nächstes anstehenden Modul eine geeignete (Rotations‑)Stelle ausfindig machen. Ein derartiges Portal ermöglicht es auch, dass die Kliniken in anonymisierter Form die Prüfungsergebnisse der teilnehmenden Weiterbildungsassistenten einsehen. Weiterhin sollen die anonymisierten Bewertungen der Ausbildungsklinik durch die Assistenzärzte ebenfalls für alle teilnehmenden Parteien einsehbar sein. Auf diese Weise kann es für den Weiterbildungsassistenten selbst, aber auch für die Ausbildungsklinik ein Feedback über den aktuellen Wissensstand und mögliche Wissenslücken geben. Umgekehrt erhalten die Weiterbildungskliniken und Praxen auf diese Weise Input bezüglich möglicher Verbesserungen an ihrer Ausbildungsstätte, die zu stetigen Bestrebungen nach Weiterentwicklung anhalten sollen, denn entsprechende Bewertungen könnten die Wahl zukünftiger Rotanten beeinflussen. Auf diesem Portal der DGU sollen den teilnehmenden Kliniken und Praxen zudem unterstützende Informationsmaterialien und Musteranträge zur Verfügung gestellt werden, um die Logistik zu erleichtern.

Als Startzeitpunkt für das neue DGU-Weiterbildungscurriculums ist der 01.01.2021 geplant. Zu diesem Datum soll es allen Weiterbildungsassistenten an teilnehmenden Praxen und Kliniken möglich sein, unabhängig von ihrem derzeitigen Ausbildungsstand in das Curriculum einzusteigen.

Nach erfolgreicher Teilnahme und Absolvierung des Curriculums erhalten alle Teilnehmer (Kliniken, Praxen und Weiterbildungsassistenten) ein anerkanntes DGU-Zertifikat. Als grundsätzliche Eignungsvoraussetzung für die Teilnahme sollten alle Weiterbilder und alle Partizipanten DGU-Mitglieder sein.

## Diskussion

Das Fehlen eines strukturierten Ausbildungscurriculums für die Facharztweiterbildung für Urologie in Deutschland wurde in der Vergangenheit häufig thematisiert [3–5]. 70 % der befragten Weiterbildungsassistenten gaben an, dass an ihren Kliniken keine curriculare Ausbildung angeboten werden würde [4].

Die European Association of Urology (EAU) veröffentlichte 2011 einen Artikel über das von ihnen neu implementierte Curriculum für eine homogenere, objektivere und verlässlichere Weiterbildung zum Facharzt für Urologie in Europa [6]. Sie betonten, dass es sich dabei weder um ein Handbuch für die Ausbildung eines Urochirurgen, noch um einen Lehrplan mit allen detaillierten Inhalten des urologischen Fachgebiets handeln solle, sondern um eine übergeordnete Empfehlung zur strukturierten Ausbildung von Weiterbildungsassistenten [6]. Ein Vorstoß der DGU zur Vereinheitlichung der deutschen Facharztweiterbildung nach diesem Vorbild, wie von zahlreichen europäischen Ländern bereits praktiziert, wurde von der Bundesärztekammer abgelehnt [2]. Deshalb entschied die DGU, ein zertifiziertes Weiterbildungscurriculum, ebenfalls im Sinne einer überregionalen Empfehlung, zu erarbeiten und Unterstützung bei der Ausführung und Koordination für alle Akteure anzubieten.

Hierbei liegt der Fokus insbesondere auf einem Rotationsprogramm, das zum einen der klaren Verlagerung vieler Weiterbildungsinhalte in den ambulanten Sektor gerecht wird und zum anderen individuelle Konzepte für die Weiterbildungsassistenten gemäß ihren Interessen ermöglicht. Rotationskonzepte wurden in der Vergangenheit schon lange diskutiert, um die intersektorale Verbundsweiterbildung zu fördern. Mit der neuen MWBO wird es zunehmend schwieriger für Einzelakteure werden, alle Weiterbildungsinhalte abzudecken und damit die volle Weiterbildungsermächtigung erhalten zu können [2]. Umso wichtiger wird es deshalb sein, entsprechende Kooperationen zwischen Universitätskliniken/Kliniken mit voller Weiterbildungsermächtigung, Schwerpunkt‑/Regelversorgern und Praxen zu bilden und so die volle Weiterbildung im Verbund anbieten zu können [7]. Insbesondere für Schwerpunktthemen wie beispielsweise Kinderurologie oder Urogynäkologie scheinen solche Rotationen sinnvoll, da die entsprechenden Weiterbildungsinhalte an vielen Häusern gar nicht mehr vermittelt werden können [5].

Auch die operative Ausbildung und die Förderung der entsprechenden Handlungskompetenzen sollte zukünftig besser umgesetzt werden. Eine Studie von Arnold et al. [4] hatte ergeben, dass 43 % der in der Studie befragten Assistenzärzte operative Eingriffe bescheinigt wurden, die sie selbst nicht durchgeführt hatten. Die Urochirurgie ist ein hochkomplexes Fachgebiet, das sich im Laufe der letzten Jahre stark weiterentwickelt und viele Subspezialisierungen hervorgebracht hat. Neue Operationsmethoden und Techniken, komplexe Eingriffe, sowie die immer fortschrittlicheren Geräte erfordern Erfahrung und hohe Kompetenz, die nur durch Training und eine gewisse Frequenz erlangt werden können. Eine fundierte und qualitativ hochwertige operative Ausbildung der Assistenzärzte soll fundamentaler Bestandteil des Curriculums sein. Auch diejenigen Assistenzärzte, die sich später im niedergelassenen Bereich betätigen wollen, sollten mögliche Operationsmethoden kennen, sich mit Komplikationsmanagement auseinandergesetzt und ein gewisses Grundrepertoire an Eingriffen (entsprechend der Empfehlung der MWBO) selbst durchgeführt haben, um Ihre Patienten später adäquat und zeitgemäß über ihre Optionen beraten zu können. Die Bescheinigung nicht durchgeführter Eingriffe und somit operativer Handlungskompetenz zum Erlangen der Facharztreife kann in der heutigen Zeit in einem derart komplexen operativen Fachgebiet nicht zielführend sein. Das Curriculum in Kombination mit dem E‑Logbuch sollen Transparenz ermöglichen, sowie den Teilnehmern die entsprechende (operative) Ausbildung garantieren.

Wie unterschiedlich die Wahrnehmung bezüglich des Vorhandenseins einer curricularen Weiterbildung von Schwerpunktthemen ist, zeigte eine Untersuchung von Kranz et al. [8], in der die Weiterbildungsassistenten angaben, zu maximal 50 % eine standardisierte urogynäkologische Ausbildung in der eigenen Klinik zu erhalten. Die Chefärzte hingegen gaben an, dass nur maximal 10 % der Weiterbildungsassistenten keine strukturierte urogynäkologische Ausbildung erhalten würden [8].

In einer Umfrage unter niedergelassenen Kollegen hatte sich gezeigt, dass nur 41 % der niedergelassenen urologischen Kollegen im Besitz einer Weiterbildungsermächtigung sind und nur etwas mehr als die Hälfte davon (24 %) tatsächlich einen Weiterbildungsassistenten beschäftigen. Als Hauptproblem wurde die Finanzierung genannt [9].

Eine kürzlich durchgeführte Untersuchung zeigte, dass in vielen Bundesländern bereits Möglichkeiten zur finanziellen Förderung und Unterstützung von urologischen Weiterbildungsassistenten in der Niederlassung (teils durch die regionalen Kassenärztlichen Vereinigungen, teils durch Fördergelder nach § 75a SGB V) bestehen [10]. Auch wenn dies noch nicht flächendeckend umgesetzt werden konnte, so zeigt sich dennoch ein klarer Trend in Richtung einer Zunahme solcher Angebote. Ein Ausbau der finanziellen Fördermöglichkeiten auf bundesweite Angebote ist sicherlich wünschenswert und ein zukünftiges Ziel der DGU.

In vielen europäischen Ländern sind sowohl die Rotation durch verschiedene Ausbildungsstätten, als auch das Absolvieren mehrerer Zwischenprüfungen vor der Facharztprüfung Standard [3]. Durch das zertifizierte Curriculum kommt die DGU dem Wunsch nach einer transparenteren und objektiver gestalteten Abfrage der Weiterbildungsinhalte durch eine schriftliche Prüfung zum Ende jedes Moduls im Rahmen der Fortbildungsveranstaltungen nach [5]. Durch die ebenfalls über eine Online-Plattform für alle Teilnehmer zugängliche Evaluation der Weiterbilder soll eine ständige Verbesserung der Weiterbildung in allen Ausbildungsstätten angeregt werden.

## Schlussfolgerung

Mit der Novellierung der MWBO hat es sich die DGU zur Aufgabe gemacht, die Weiterbildung in Deutschland im Rahmen der gesetzlichen Möglichkeiten homogener, transparenter und strukturierter zu gestalten. Durch das neue DGU-zertifizierte Weiterbildungscurriculum kommt sie den Anforderungen nach, die an eine moderne Weiterbildung nach EU-Vorbild gestellt werden. Die Implementierung des Curriculums erfordert Offenheit für Veränderung, Flexibilität und ein Umdenken bei den Akteuren. Voraussetzung für die erfolgreiche Umsetzung des Curriculums ist der Wille, mit den Zeichen der Zeit zu gehen und die durch die Novellierung der MWBO entstandene Chance zur Verbesserung der Weiterbildung zu nutzen. Wenn dies gelingt, so kann das DGU-zertifizierte Weiterbildungscurriculum künftig ein Gütesiegel für urologische Weiterbildung in Deutschland darstellen.
